# Tissue engineering in burn scar reconstruction

**DOI:** 10.1186/s41038-015-0017-5

**Published:** 2015-09-30

**Authors:** PPM van Zuijlen, KLM Gardien, MEH Jaspers, EJ Bos, DC Baas, AJM van Trier, E Middelkoop

**Affiliations:** 1 Burn Center, Red Cross Hospital, Beverwijk, The Netherlands; 2 Department of Plastic, Reconstructive and Hand Surgery, Red Cross Hospital, Beverwijk, The Netherlands; 3 Association of Dutch Burn Centers, Beverwijk, The Netherlands; 4 Department of Plastic, Reconstructive and Hand Surgery, MOVE Research Institute, VU University Medical Center, Amsterdam, The Netherlands

**Keywords:** Burns, Tissue engineering, Scar, Contracture, Substitute, Subcutaneous fat tissue, Cartilage, Reconstruction

## Abstract

Nowadays, most patients with severe burns will survive their injury. This evolution is accompanied by the challenge to cover a large percentage of total body surface area burned. Consequently, more and more patients have to deal with the sequelae of burn scars and require (multiple) reconstructions. This review provides a gross overview of developments in the field of tissue engineering for permanent burn wound coverage and reconstructive burn surgery, focusing on usage and clinical effectiveness. Not only skin substitutes will be discussed but also the replacement of subcutaneous fat tissue and cartilage.

## Introduction

Reconstructive surgery has been performed since ancient times, especially circumstances such as disasters and wars frequently challenge humanity to innovate. Pioneers like Sir Harold Gillies and Sir Archibald McIndoe have developed many techniques for the reconstruction of all sorts of traumatic wounds during the First and Second World Wars. During the last World War, many young pilots crashed with their airplane when it was hit by enemy fire. Some of them survived the crash and were able to return to England, but many of them sustained severe burn injuries to the hands and faces. McIndoe treated many of these war survivors in his hospital in East Grinstead. He did remarkable work and invented many interesting new flaps and techniques for burn scar reconstruction. However, at that time, the survival of patients was poor, especially of those with extensive burn wounds.

Since then, significant progression has been made concerning the resuscitation and emergency management of severely burned patients. Most patients with extensive burns will survive their injuries nowadays. This necessitated the development of techniques to close wounds of more than half the body surface area. The autologous split thickness skin graft, which is an epidermal graft with only a thin dermal layer, became the mainstay of burn surgery because of donor site availability and of the potential for reuse of the same site after a couple of weeks. Despite the importance of this development, this only solved part of the problem.

Expansion techniques such as mesh grafting and Meek Wall micrografting were developed to improve the efficacy of autologous split skin grafting [1, 2]. At that time, closing of the wound, which practically meant restoring the epidermis, was the main focus. This explains why Rheinwald and Green explored the possibility of culturing keratinocytes to create the first epidermal substitutes simultaneously in the early 70s [3, 4]. All these techniques contributed indisputably to closure of extensive burn wounds mainly by restoring the epidermal layer.

Unfortunately, the grafted areas were known to result in a scar with a poor esthetic result with functional problems such as contracture and stiffness. Also, many location specific complaints, such as ectropion of the eyelid, nasal obstruction, oral incontinence, swan neck/boutonniere deformity of the finger, and claw hand may result in scar formation. Less functional problematic but also disfiguring are erythema, aberrant pigmentation, and textural deformities. Pain and mostly itching are “complaints in disguise” that can be extremely disturbing for the patient affecting their quality of life [5].

So the closed wound has become a scar that mainly lacks “dermal qualities” such as flexibility and strength. However, it is misleading to conclude that thicker split thickness skin grafts should be used to provide the best outcome in terms of scar formation because donor sites heal more problematic after harvesting thicker split thickness grafts. Certainly in extensively burned patients, where donor sites may have to be used several times, it is therefore advised to use thin split skin grafts because these donor sites will heal quickly and spontaneously.

In the 1970s, it was already concluded that there is not sufficient autologous dermis available for transplantation of large defects. This challenged the surgeons and researchers to engineer dermal like tissue in the lab. Tissue engineering of a dermis seemed the logical choice. Since then, many studies on dermal substitution have become available. Products have been developed and some, like Integra and MatriDerm, have become commercially available and proved to be a valuable addition to the armamentarium for burn wound treatment. Table 1 provides an overview of a selection of commercially available skin substitutes.

**Table 1:** A selection of commercially available skin substitutes

Product	Manufacturer	Basic composition
Bilayered (dermo-epidermal) substitutes		
Apligraf ®	Organogenesis Inc., MA, USA	Bovine collagen I gel with allogeneic human keratinocytes and fibroblasts
OrCel ™	Forticell Bioscience Inc., NY, USA	Bovine collagen sponge with allogeneic fibroblasts and keratinocytes
Epidermal substitutes		
Epicel ®	Genzyme Corporation, Cambridge MA, USA	Cultured substitute: autologous keratinocytes with murine fibroblasts
Laserskin ®	Fidia Advanced Biopolymers, Abano Terme, Italy	Cultured substitute: benzyl esterified hyaluronic acid derivative with autologous keratinocytes
Dermal substitutes		
Matriderm ®	MedSkin Solutions Dr. Suwelack AG, Billerbeck, Germany	Acellular bovine dermis
Alloderm ®	LifeCell Corporation, Bridgewater, NJ, USA	Acellular human dermis
Integra ®	Integra Life Sciences Corp., NJ, USA	Bovine collagen and glycosaminoglycan with silicone top layer
Oasis ®	Smith & Nephew Inc. Fort Worth, TX, USA	Porcine small intestine submucosa
Glyaderm ®	Euro Skin Bank (ESB), Beverwijk, The Netherlands.	Acellular human dermis
Permacol ™	Covedien, Mansfiels, MA, USA	Acellular porcine dermis
Graftjacket ®	Wright Medical technology Inc., Memphis, TN, USA	Acellular human dermis
Dermagraft ®	Organogenesis Inc., MA, USA	Allogeneic human fibroblasts and polyglactin mesh scaffold
EZ Derm ®	Mölnlycke Health Care AB, Gothenburg, Sweden	Porcine collagen dermis

Overview of commercially available epidermal, dermal, and complete skin substitutes for wound repair

This review provides a gross overview of tissue-engineered products developed for permanent burn wound coverage and scar reconstruction. It will mainly concern epidermal and dermal substitutes, but it will also clearly extend its view beyond the skin because subcutaneous fat and cartilage are becoming more interesting from a tissue engineering perspective.

Nowadays, the role of the subcutis as a gliding layer for the scarred skin has been well appreciated. Burn scar reconstruction clearly also focuses on restoring the subcutaneous layer. At the same time, tissue engineering of cartilage became of interest for reconstruction of ears and nose after severe facial burns.

## Review

### Tissue engineering

The term “tissue engineering” is associated with the repair of body tissues making use of cells, artificially created lattices and/or specific biochemical substances. Stem cells or progenitor cells can also be used to produce tissues. Twenty years ago, these three strategies for the creation of new tissue were already described by Langer and Vacant (i.e., isolated cells or cell substitutes, tissue-inducing substances, and cells placed on or within matrices) [6]. Tissue-engineered constructs like cartilage, blood vessels, bladder, skin, and muscle are now being created in the lab and some have clinical applications.

Some tissues, like the epidermis, consist predominantly out of cells and can be engineered by cell culturing techniques. But other tissues, like cartilage, are mainly made of an extracellular matrix that provides 3D form and mechanical properties to the tissue. The matrix also serves as a template for the cells (chondrocytes). Ear cartilage is a good example where structure and function are of uttermost importance. Engineering of ear cartilage without providing a sustainable 3D structure with appropriate elasticity is not clinically relevant. Thus, a template has to be fabricated in optimal 3D ear form which meanwhile supports the cells and their function in creating and maintaining their normal extracellular matrix. This is an interesting topic at the moment where tissue engineering and 3D printing are merging.

An overview is given below on developments in the field of tissue engineering concerning reconstructive burn surgery. Therefore, not only skin substitutes will be discussed but also the replacement of subcutaneous fat tissue and cartilage. This review focuses on permanent substitutes and not on products engineered for temporary replacement, such as tissue-engineered wound dressings based on allogeneic cells.

#### Epidermal substitutes

Rheinwald and Green were the first to report a successful method to culture keratinocyte colonies from single-cell suspensions of human epidermal cells in 1975 [4]. This technique was potentially life-saving at that time as it could facilitate wound closure for patients that survived extensive burn wounds especially when donor site availability was limited. Since then, cultured epithelial autograft (CEA) became available to be used as confluent sheets to provide permanent burn wound coverage. Six years after Rheinwald and Green’s publication, the first clinical application was reported [7]. The technique has been used worldwide for many cases.

Clinical studies demonstrated variable successes; a major concern was the poor survival of the keratinocyte sheets [8]. A meta-analysis on graft survival showed a poor survival of about 60 %. The main causes of graft failure were wound infection and hemorrhage [9, 10].

In 2007, Atiyeh et al. reviewed the successes and problems of CEA for burn treatment in more than three decades [11].

Besides its potential as a method to provide permanent wound closure for extremely burned patients, the weaknesses of this treatment also became obvious. Its widespread use has been hampered by delays caused by the time necessary to culture enough keratinocytes to cover extensive burn wounds. Obviously, large expansion rates are required for extremely burned patients and culturing cells into sheets then may take 3 or 4 weeks. This can be problematic from a clinical perspective. Another frequent criticism of CEA is their extreme fragility and susceptibility for bacterial contamination. Atiyeh concluded that the initial optimism for cultured keratinocyte grafts has gradually declined. In the long term, easy blister formation of the treated area was noted because of a lack of formation of anchoring filaments at the level of the basement membrane.

Others also stressed the uncertainty of the take rate, the high cost, and the problematic scarring in deep burn injuries because of the lack of dermis [12].

There are several commercially available epidermal substitutes available; most of them are applied for acute burn wound treatment. Unfortunately, there is still a lack of randomized controlled trials, and the use of good outcome parameters is limited [13]. To circumvent such problems such as the reduced take rate and the critical delay in time to produce multi-layered keratinocytes sheets, new delivery systems have been developed to transfer keratinocytes.

The development of biocompatible carriers for single-layered keratinocytes allowed grafting at earlier culturing stages [14, 15]. The concept of using pre-confluent keratinocytes, where colonies of keratinocytes are grafted onto the wound bed before they form a sheet, has also been used [11].

Another option is to prepare an epidermal cell spray directly during surgery. This can be done on site by special kits that are developed for this purpose, such as ReCell (Avita Medical Europe Ltd, Melbourn, UK,). Usually a thin split thickness skin graft is harvested because this allows better separation of the epidermal layer from the dermal layer, facilitating the isolation of epidermal cells. This can be useful for indications of small burns where no culturing time is required. However, Gravante et al. compared the ReCell procedure with the standard transplantation of autologous skin grafting and concluded that the ReCell procedure took more time and thus costs (operating room) without real benefit regarding functional and esthetic outcome [16]. Nevertheless, they concluded that this product could reduce the need of donor sites in deep dermal injuries and that the spared donor sites can be used to cover full-thickness wounds. In another recently published study, ReCell was also compared with autologous meshed split thickness skin graft for acute burn injuries and found to be beneficial for the patients because of a decreased donor site size with comparable outcome. The cell spray method can also be used for the correction of pigment disorders, as melanocytes are also included in the cell suspension that is sprayed onto the wound [17, 18].

Moreover, the combination of abrasion and the cell spray procedure may allow a more aggressive treatment of a disturbed texture, which is frequently observed after burn injuries.

#### Dermal substitutes

The publications by Yannas and Burke on the basic physical requirements of dermal substitutes heralded the start of a new area in wound healing [19]. Following their principles, they developed a dermal substitute with a silicon layer on top that serves as a temporary epidermal cover. It became commercially available as Integra Dermal Regeneration Template (LifeSciences Corp., Plainsboro, NJ, USA), and it is currently being used for acute burns and scar reconstruction.

Various dermal substitutes have been developed like collagen scaffolds, synthetic materials, or cadaveric skin. These substitutes are being combined with cells, mostly fibroblasts, to create a living dermal substitute. Experimental studies showed that dermal regeneration can be improved and wound contraction can be reduced with fibroblasts [20–22]. Both allogeneic and autologous fibroblasts have been used, and it was demonstrated that they are beneficial with respect to macroscopic as well as histopathological results in different *in vitro* and animal studies [21–25].

In a clinical study, it was shown that TransCyte® (Smith&Nephew plc, London UK), a bioabsorbable polyglactin mesh seeded with allogeneic neonatal fibroblasts, promotes re-epithelialization when used in partial-thickness burns in children [26]. This product, however, has only been approved as temporary skin replacement. Obviously, allogeneic cells will finally be rejected due to immune reactivity of the recipient [27, 28]. Allogeneic fibroblasts are not being used in products developed for permanent skin replacement. The major drawback of autologous fibroblasts is the delay in grafting that is caused by the time required to culture sufficient cells.

This probably explains why acellular dermal substitutes are still the most popular for clinical application. Besides Integra, MatriDerm is now being used worldwide on a large scale. Both are “off the shelf” substitutes that make them more practical with fewer costs for manufacturing. Interestingly, both are based on an engineered collagen scaffold.

##### Integra

Integra is a dermal substitute consisting of bovine collagen and chondroitin 6-sulfate covered by a disposable “epidermal” silicone layer. The silicone sheet acts as a barrier against bacteria and water evaporation; moreover, it provides mechanical support. During the first operation of this two-staged procedure, the bilayered membrane is applied. After 2 to 3 weeks, the silicone layer will be removed and replaced by a thin split skin graft that serves as an epithelial graft. In the first clinical study, a good neodermis was provided resembling the normal dermis [29].

Integra might be considered for extensive acute burns mainly because it allows early removal of the eschar, and it provides direct wound coverage. Therefore, it reduces the need for donor sites in the beginning, which can be crucial for the optimal final treatment. Heimbach et al. published a multicentre trial on Integra on a large cohort of patients [29]. After 1 year, less hypertrophic scarring was noted with similar appearance and function compared to the control site. In addition, histopathological studies confirmed that patients treated with Integra demonstrated good wound repair with a minimum of scarring [30, 31]. The long-term results range from normal to notable supple scar tissue. It leaves a smooth scar where the interstices of the meshed split thickness skin graft are hardly visible.

If Integra is used for the trunk and extremities in an extensively burned patient, it can reduce the need for donor sites significantly at the start of treatment. A severely burned face is also an important indication because of the final appearance. Integra should then preferably be applied as one unit if possible. If more than one unit has to be used, it has been advised to apply Integra not according to the esthetic units (because the skin grafts also have to be situated in this way).

Moreover, severe hand burns are also being treated with success [32–35]. In a study involving 216 burn injury patients who were treated at 13 burn care facilities in the USA, the safety and effectiveness of Integra was evaluated [36]. Heimbach et al. showed that the mean take rate of Integra was 76.2 % with a median take rate of 95 %. This shows that loss of Integra should be anticipated. The mean take rate of epidermal autografts was 87.7 % with a median take rate of 98 %. The incidence of invasive infection at Integra-treated sites was 3.1 % and that of superficial infection 13.2 %. Muangman et al. demonstrated that Integra might perform well despite high-bacterial counts if wounds are treated with appropriate topical and systemic antibiotics [37]. Lohana et al. reported overall satisfactory results to both patient and surgeon regarding pliability, final function, and cosmetic appearance despite the commonly observed infection at the graft site [38].

The superior results in scarring make Integra also feasible for scar reconstructions. Problematic scars can be completely removed and resurfaced by Integra [39]. After 2 to 3 weeks, the silicon layer is removed and replaced by a very thin split thickness autograft (preferably as full sheet). The results are good with respect to scarring as it normally results in supple, normotrophic scars [40]. Without the interposition of Integra scar formation would probably have been worse if a thin split skin graft was used. Does this prove that Integra is better for this indication? There is definitely a need for well-designed comparative, randomized, and blinded studies. But this is practically infeasible and sometimes even unethical. So far many have demonstrated superior results on the use of Integra for scar reconstructions in clinical studies [41–43]. Integra has also been applied for breast and hand reconstruction with favorable cosmetic and functional outcomes [34, 44]. Moreover, it also appears to have a role in the reconstruction of complex defects with exposed bones and tendons. Many reports are published on the successful use of Integra for this type of difficult wounds [35, 37, 45, 46]. Integra can also be used in contracture release procedures [47, 48]. It has been used in the neck, axilla, elbow, knee, hand, and other anatomical sites. Frame et al. reported on a large cohort of contracture releases in multicenter investigation. Approximately 75 % of the release sites showed a significant improvement in range of motion or function. But in 25 %, recurrence of contracture was observed during follow-up monitoring [48]. Others also reported that Integra sometimes shows re-contraction of the scar when applied in the neck area [49]. Furthermore, the results in children sometimes seem disappointing because Integra could not reduce the need for reconstructive surgery [50–52]. In both situations, the “Integra-scar” apparently could not anticipate the continuous mechanical forces sufficiently.

To conclude, there is certainly a beneficial role of Integra in acute burn surgery and scar reconstruction. In acute burns, it could lead to a reduced initial need for donor sites while it improves the quality of scarring. Then Integra may be life-saving. For scar reconstruction, many have demonstrated a superior outcome of scarring in relation to the use of Integra. However, re-contraction of Integra may occur when used for scar contracture releasing procedures.

##### Matriderm

MatriDerm (MedSkin Solutions Dr. Suwelack AG, Germany) is a single layer dermal substitute that consists of bovine collagen and an elastin hydrolysate. The first clinical study was published in the year 2000 and reported on the use of this material for acute burn wounds and scar reconstruction procedures. It was a comparative study design where the dermal substitute was applied together with a split skin graft and compared to the outcome of a split skin graft alone [53]. Autograft survival was not altered by the substitute for reconstructive wounds, but a 9 % reduction of the take rate of the autograft was established in the burn category for substituted wounds compared with non-substituted wounds. However, the necessity for re-grafting was not increased by substitution [53]. Therefore, it was concluded that this product could safely be applied in a one-step procedure. Objective and subjective scar assessment tools such as elasticity measurements, colorimetry, and the Vancouver Scar Scale were used for the analysis. A significant increase in elasticity of scar tissue was noted 3 months after scar reconstructions. For acute burn wounds, this increase in elasticity was not established. It was noted clinically that the surface roughness and texture of the areas with dermal substitution seemed superior. Unfortunately, it was not possible to analyze this parameter objectively at that time.

After 12 years, the same cohort was evaluated again [54]. A long-lasting beneficial effect on scar quality was demonstrated [54]. At that time, it was also possible to perform an objective analysis of surface roughness. Subjective assessment in acute and reconstructive burn scars by means of the POSAS, the Patient and Observer Scar Assessment Scale, showed several statistically significant differences in favor of substituted scars [55–57]. The analysis included pliability, relief, and the general observer score of the POSAS. Elasticity measurements showed higher scores for substituted scars, although the difference was not statistically significant. Probably, the most interesting finding was observed in the category of acute burn wounds. At first sight, no benefit of the dermal substitute could be noted in this group. However, when an analysis was performed of those wounds which were treated with largely expanded meshed skin grafts, a significantly higher elasticity was found for the substituted area compared to the control area. This finding is also clinically significant because the application of dermal substitutes is particularly warranted in severely burned patients when donor sites are sparse and widely expanded grafts are inevitable.

Later, Bloemen et al. performed a four-armed multicenter randomized controlled trial, where a split skin graft with or without a dermal substitute and with or without topical negative pressure was compared in patients with deep dermal or full-thickness burns requiring skin transplantation [58]. A graft take of more than 90 % was found in all categories. An improved effectiveness of the dermal substitute was found when combined with topical negative pressure. In another study (concerning Integra), Moiemen could not demonstrate increased neovascularisation by negative pressure therapy [59].

Hur et al. studied the progress of skin graft maturation through measuring the size of the scar in comparison with the original wound area [60]. They showed that grafted skin underwent contracture and remodeling for 3–6 months. Grafts on acute burns showed more contraction than reconstructive defects. However, the use of MatriDerm reduced the contracture rate and enhanced skin elasticity although the contracture rate could not be eliminated completely by using this dermal substitute [60, 61]. Many other studies have been performed for acute burn wounds and scar reconstructions. Most studies find a favorable effect of MatriDerm [62–64]. MatriDerm has been frequently applied and tested specifically for hand burns and reconstructions in that area with positive results [65–68]. Demircan et al. concluded that the esthetic and functional results of MatriDerm in children were encouraging [69].

MatriDerm can be applied successfully in a one-step procedure. This can be an advantage of MatriDerm over Integra in many burn cases, especially in scar reconstruction. However, well-designed clinical studies comparing MatriDerm and Integra for this purpose are lacking. In a rat model, however, no major differences in engraftment rates or vascularisation were found when comparing MatriDerm with Integra [70]. In a porcine full-thickness wound model, five dermal substitutes were compared. No long-term difference of scar quality between the different substitutes and the control group was seen. The authors therefore question the benefit of a two-step procedure [71]. Nevertheless, we clearly see a role for a two-step procedure making use of the bilayered version of Integra in treating extensively burned patients. Integra is also available as a single layer now. It has been tested in a rat model showing that both templates contain a comparable biological behavior early after transplantation. The only differences found were the faster degradation of MatriDerm and a difference in neodermal thickness [72].

##### Allograft

Allograft can be used as a temporary as well as permanent skin replacement. Freeze-dried or glycerolized allograft is frequently used as a biological wound dressing especially in many burn centers where early excision of the burn eschar is done routinely. Dressing changes with allografts are performed until definitive wound coverage is undertaken.

Allograft can also be used as a permanent replacement for the dermis because it contains natural collagen, elastin, and basement membrane structures. Cuono et al. reported clinical cases on successful skin resurfacing with an allogeneic cryopreserved dermis engrafted with cultured epidermal autografts [73, 74]. The non-cellular components of the cadaveric dermis have been shown to be relatively non-immunogenic, but the epidermis cannot be used as permanent coverage because of its antigenicity. AlloDerm® Regenerative Tissue Matrix (LifeCell Corporation, Bridgewater, NJ, USA) is a commercially available product that was tested for scar reconstruction purposes. Burn scar contractures and depigmented areas of the upper extremity were improved by a combination of dermabrasion and an AlloDerm graft over the scar-releasing defect [75]. There are not many studies performed on AlloDerm or other allografts in the last decade [76]. Recently, a study has been published on a glycerol preserved acellular dermal replacement product named Glyaderm (Euro Skin Bank, Beverwijk, The Netherlands), which was demonstrated to serve as a dermal substitute in acute burn wound surgery [77]. The combination of Glyaderm^®^ and a split thickness autograft was superior to only a split thickness autograft at 1 year after surgery, regarding elasticity measurements and the judgement of blinded observers.

Although promising studies have been published, there remains a scarcity on the studies concerning the use of allogeneic cadaveric skin.

### Cell-based skin constructs

Much progress has been made over the past decades in the development of cell-based skin substitutes for full-thickness skin defects. The first promising clinical results with the CEAs in the 1970s contributed to further development of bilayered dermo-epidermal skin substitutes for burn treatment. Nevertheless, there are only a few products commercially available yet. Most of these products consist merely of human allogeneic skin cells. Apligraf ® (Organogenesis Inc., MA, USA) consists of human allogeneic keratinocytes and fibroblasts derived from neonatal foreskins seeded onto a bovine type I collagen sponge [78–81]. The FDA approved the use of this product for venous leg ulcer and diabetic foot ulcer. Another example is OrCel® (Forticell Bioscience Inc., NY, USA) in which human allogeneic epidermal keratinocytes and dermal fibroblasts are cultured into a type I bovine collagen sponge [82]. In the review of Debels et al., an overview is given of the research on the bilayered dermo-epidermal autologous cultured skin substitute PermaDerm™ (Regenicis Inc., NY, USA), which is composed of autologous cultured keratinocytes and fibroblasts onto an absorbable collagen substrate (biomedical polymer) [83].

However, the same clinical implementation issues as for the CEAs apply to the cell-based bilayered skin substitutes. Well-known examples are the long culture time, limited tolerance of mechanical friction, high costs (e.g., production, storage, laboratory facilities, transport), and regulatory requirement issues.

A tremendous effort has to be made by researchers and clinicians to fulfill all the regulatory issues that accompany the clinical application of an autologous cell-based substitute. As a result, the evidence level of these promising cell-based products is still limited.

In order to create and replace the complete structure of the skin with the cosmetic appearance and all the characteristics of this complicated organ, also other important structures such as blood vessels, nerves, hair follicles, sweat glands, and subcutaneous adipose tissue are required. Most studies in this field are still experimental preclinical studies. To achieve vascularization in a skin substitute, different methods are currently available and a description of these approaches is outlined in a review of Auger et al. [84]. The regeneration of nerves is complicated, and to our knowledge little is published about the implementation of nerve regenerative cells in dermal substitutes. Hair follicle development in dermal skin substitutes has been studied about the differentiation of stem cells [85]. The ability of preadipocytes to proliferate and differentiate after transplantation into adipose tissue is essential to develop soft tissue in the engineered skin. To create the subcutaneous layer of the skin, research is performed to culture human preadipocytes in scaffolds [86, 87]. Keck et al. reported on simultaneous culturing of human preadipocytes and keratinocytes onto a collagen-elastin scaffold [86]. Trottier et al. described in vitro production of skin substitutes reconstructed from keratinocytes and adipose-derived stem/stromal cells without a synthetic scaffold [88].

Over the last few years, tissue engineering seems to reach a new era by the discovery of the availability of stem cells (SCs) [89, 90]. This new technology is of great interest and is bringing cellular therapy at the front line of the field of tissue engineering for human treatment [91]. SCs contain multilineage differentiation capacity and immune-modulating effects [92], which make them ideal for tissue-engineering purposes. A few clinical case studies have shown that SCs could ameliorate burn wound healing and even deregeneration of skin appendages is reported [93–95]. Until now, bone marrow-derived mesenchymal SCs are the most favored cell type presently under clinical trial, but the use of adipose-derived SCs is increasing. This exciting field is associated with an increasingly amount of in vitro experiments, animal models, and clinical trials [96, 97]. However, the safety for human use is not fully elucidated yet, and the culturing expenses are very high. This makes the use of SCs very promising but not common practice nowadays.

All this research on different cell types and structures is encouraging for the exploration of combinations of all these engineered tissues in one skin substitute. Over the years, it may be possible to extend tissue-engineering technologies and bioprint a 3D skin [98, 99]. All these experimental developments may be available for clinical studies and eventually provide an option for treatment modalities in patients with full-thickness skin defects.

### Fat

Until recently, burn specialists almost exclusively focused on engineering of skin. However, there is an increasing understanding of the significance of the subcutaneous layer for the mobility and quality of the skin. This explains the unequivocal increase in clinical studies on reconstruction of the subcutaneous layer and experimental studies on engineering of fat.

The important role of fat for the function of the skin becomes especially apparent in burn patients. In cases where fascial excision was necessary, scars become directly attached to underlying tissues such as fascia, bone, tendon, and muscle. The mobility of those scars is considerably hampered because the gliding layer is lacking.

Fortunately, adipose tissue can be easily harvested by liposuction and used to reverse the state of damaged tissue [100, 101]. Besides adipocytes, the harvested fat tissue contains adipose-derived stem cells (ASCs), vascular endothelial cells (VECs), fibroblasts, pericytes, and connective tissue as well as adipose tissue-resident macrophages and lymphocytes [20, 102]. The regenerative effect of autologous fat is considered to be derived from ASCs. These cells comprise the potential to release angiogenic growth factors in response to ischemic fibrous tissue and to differentiate into adipocytes and VECs [103]. Moreover, 1–2 % of ASCs have the potential to differentiate into multilineage cells (i.e., adipogenic, chondrogenic, myogenic, and osteogenic), implicating even more possibilities for adipose stem cell-based tissue engineering [104]. It is hypothesized that ASCs are the main cell population contributing to a balance between adipocyte apoptosis/necrosis and adipogenesis after autologous fat grafting [105]. This mechanism is of importance when reconstructing and restoring the subcutis, which is often destroyed in severe burns cases. As mentioned earlier, a burn scar will adhere to the underlying fascia since the important quality of the subcutis as a functional sliding layer is hampered. But besides restoring the function of the subcutis, autologous fat seems to improve the dermal quality of a scar as well. In an experimental animal study, it has been shown that fat grafting stimulates a neosynthesis of collagen fibers at the recipient site [106]. Another study emphasizes the suppression of fibrogenesis after injury by ASCs secreting growth factors [107]. Clinical studies on the effect of fat grafting in burn scars also mention favorable results in terms of pliability. Unfortunately, the conclusions were not sufficiently warranted by study design and/or the quality of the outcome parameters [108–110]. Autologous fat grafting is becoming a valuable addition to the reconstructive armamentarium in burn scars; however, long-term studies providing objective data are needed to demonstrate its effectiveness.

### Cartilage

Despite all improvements in acute burn wound treatment, a severe facial burn injury frequently will be immensely disfiguring. The esthetic and functional impact of a mutilated ear or nose is considerable. Both the ear and nose, which are only covered by a thin skin layer, are particularly vulnerable to thermal injury. Their structure and anatomy is highly related to its cartilage framework directly underneath the skin, and this cartilage does not have regenerative capacities.

In the acute phase, all exposed cartilage should be protected maximally to maintain the structure of the ear as good as possible. If necessary, a reconstruction of the ear is performed in a later phase. Reconstruction of ears and noses in burn victims are still demanding surgical procedures. It is particularly difficult due to loss of normal skin and scarring of the surrounding tissue, which does not facilitate coverage of the cartilage framework. The quality of the surrounding tissue dictates the possibilities.

Costal cartilage and synthetic permanent implants have shown their beneficial effect in ear reconstruction, but both have their disadvantages. Creating an ear form from costal cartilage is a quite demanding procedure, and it does not have the same elastic properties as ear cartilage. Synthetic permanent implants like Medpor® have a higher risk of infection and rejection depending on the quality of skin that covers the implant.

Tissue engineering of cartilage for nose and ear reconstruction after a severe facial burn injury is therefore an interesting new alternative. Individualized and customized constructs may be facilitated by the use of advanced 3D bioprinting for cartilage engineering [111]. Promising results have been obtained with experimental studies; however, the transition to clinical practice is still challenging. Tissue-engineered cartilage constructs for facial reconstruction will hopefully find their way to the clinic within several years.

## Conclusions

Patients nowadays may survive severe burn injuries because of the remarkable improvement in emergency management, shock treatment, and wound care. So many lives are being saved and extended. This calls for a paradigm shift in treating burn patients, from short term to long term, from survival to quality of life, and from tissue repair to tissue regeneration.

Some types of tissue are capable of regeneration. A good example is the epidermis; this is a highly cellular structure with many stem cells that continuously regenerates itself. Other types of tissue such as the dermal layer, the subcutaneous layer, and cartilage do not share that possibility of regeneration after wounding. A wound can become closed when epidermal remnants are present in the wound. This means that the epidermal layer is intact again; it is more or less regenerated. But it does not mean that the dermis healed well. Also, the underlying subcutaneous fat and cartilage show tremendous difficulties in repairing itself.

This is why tissue engineering is so interesting and why it has great potential in burn treatment in order to improve the quality of life by improving the outcome of burn surgery.

Since the pioneering work on epidermal and dermal replacements, four decades have passed. A critical appraisal of the literature shows that products of epidermal regeneration are scarcely used in clinical practice.

Interestingly, the acellular dermal regeneration templates like Integra, MatriDerm, and AlloDerm are quite frequently used. The number of well-designed clinical studies is low, but many reports have been published mentioning considerable success of these dermal templates. Mostly, an improvement in quality of scar tissue was shown. This positive finding paradoxically also leads to a negative conclusion; we are still creating scar tissue, and therefore, we are not regenerating tissue.

All these commercially available products are typically acellular. The implementation of cells into the engineered construct may be crucial to come to true tissue regeneration. Experimental studies on cellular constructs are sometimes very promising, but more convincing evidence from clinical studies would be appreciated. The high costs and the regulatory issues do not facilitate the transition from lab to clinical practice.

Available products like the dermal regeneration templates can be used in acute burn wounds as well as for scar reconstructions. Dermal templates can be easily used certainly if other options like local flaps or full-thickness skin grafts are not available for that specific scar reconstruction procedure. Engineering of fat and cartilage is relatively new but already promising for scar reconstruction. The introduction of advanced 3D bioprinters may have a significant role in tissue engineering, specifically for cartilage.

Hopefully, the patients will benefit more from future developments in the field of tissue engineering and regenerative medicine.
